# Core targets of bisphenol A in cervical cancer revealed by network toxicology and molecular docking

**DOI:** 10.1097/MD.0000000000047993

**Published:** 2026-03-06

**Authors:** Yi Li, Wanjun Zhang, Lin Tang, Yuqian Zhu, Hongli Dong, Long Yang, Yutai Zhao

**Affiliations:** a Department of Gynaecology and Obstetrics, Ya’an People’s Hospital, Ya’an, Sichuan Province, People’s Republic of China; b Department of Gynaecology and Obstetrics, The First Affiliated Hospital of Chengdu Medical College, Chengdu, Sichuan Province, People’s Republic of China.

**Keywords:** bisphenol A, cervical cancer, ESR1, molecular docking, network toxicology, PARP1

## Abstract

This study aimed to investigate the molecular mechanisms underlying BPA(Bisphenol A)–induced cervical cancer, to identify core targets and signaling pathways, and to provide a theoretical basis for disease prevention and therapeutic intervention. The chemical structure of BPA was obtained from PubChem(Public Chemical Database), and its toxicity profile was evaluated using ProTox-3.0. Potential BPA-associated targets were predicted using multiple databases and subsequently standardized. Differentially expressed genes (DEGs) in cervical cancer were identified from Gene Expression Omnibus datasets using the R programming language and integrated with Weighted Gene Co-expression Network Analysis (WGCNA) to determine key module genes. The cervical cancer-related target set was then established. Common targets between BPA and cervical cancer were identified using Venn diagram analysis, and a protein–protein interaction (PPI) network was constructed to screen for core targets. Gene Ontology and Kyoto Encyclopedia of Genes and Genomes enrichment analyses were performed to explore biological functions and pathways. Target gene expression was validated across multiple datasets, and molecular docking analysis was conducted using Cavity-Based Dock 2 (CB-Dock2). BPA exhibits endocrine toxicity and matrix metalloproteinase-mediated tissue damage, with 3 core targets identified across databases. In cervical cancer, 803 up-regulated and 1092 down-regulated DEGs were screened (|log_2_FC| ≥1, adjusted *P* <.05). WGCNA identified the turquoise module (normal group *R* = 0.98, *P* = 5 × 10^−12^; cancer group *r* = −0.98, *P* = 5 × 10^−12^), overlapping with 1110 DEGs. Nineteen common targets of BPA and cervical cancer were enriched in gene expression negative regulation and cancer pathways (hypergeometric test, false discovery rate (FDR) <0.05). PPI analysis confirmed Estrogen Receptor 1 (ESR1) and (Poly [ADP-ribose] Polymerase 1 (PARP1) as core targets: ESR1 was down-regulated (GSE122697: log_2_FC = −2.8, *P* <.0001; The Cancer Genome Atlas (TCGA): log_2_FC = -2.6, *P* <.0001), PARP1 up-regulated (GSE122697: log_2_FC = 3.1, *P* <.0001; TCGA: log_2_FC = 2.9, *P* = .0012). Both showed progressive expression changes with lesion advancement (GSE63514: ESR1 log_2_FC = -4.2, PARP1 log_2_FC = 4.5, *P* <.0001). Molecular docking revealed stable binding of BPA to ESR1 (−8.3 kcal/mol) and PARP1 (−8.5 kcal/mol, root-mean-square deviation [RMSD] <2.0 A). BPA may promote cervical carcinogenesis by interacting with ESR1 and PARP1 to regulate key cancer-related pathways. These targets may serve as potential biomarkers and therapeutic intervention points. Further experimental validation is required to confirm these findings.

## 1. Introduction

Cervical cancer is the fourth most common malignancy among women worldwide, with a disease burden that varies markedly across different regions.^[1,2]^ According to the World Health Organization, approximately 660,000 new cases of cervical cancer and 350,000 related deaths were reported globally in 2022, with more than 94% of these deaths occurring in low- and middle-income countries.^[3,4]^ In China, cervical cancer ranks second among gynecologic malignancies in terms of incidence. In 2020, there were 109,741 newly diagnosed cases (accounting for 18.2% of global cases) and 59,060 deaths (17.3% of global deaths).^[5]^ Notably, the incidence of cervical cancer in rural areas of central and western China is significantly higher than that in urban regions of eastern China, making it a major public health concern that poses a serious threat to women’s health. The disease also exerts a profound social impact; approximately 20% of children lose parental care due to maternal death from cervical cancer, further exacerbating health inequities.^[6,7]^

In recent years, increasing attention has been directed toward the role of environmental endocrine disruptors (EDCs) in the development of cervical cancer.^[8]^ BPA, a ubiquitous environmental estrogen widely used in plastic products and food packaging materials, can accumulate in living organisms, mimic endogenous estrogen activity, and disrupt endocrine homeostasis. Growing evidence suggests that chemical exposure–induced damage to the uterine cervix may contribute to adverse reproductive outcomes. Moreover, global exposure to BPA has increased steadily across different regions in recent decades.^[9,10]^

Network toxicology is an emerging interdisciplinary field that integrates systems biology, bioinformatics, and big data analytics to elucidate the complex interactions between environmental pollutants and human diseases.^[11]^ The core concept of network toxicology is the construction and analysis of a systematic “pollutant–target–pathway–disease” interaction network to clarify the molecular mechanisms by which environmental toxicants induce disease.^[12]^ This approach overcomes the limitations of traditional toxicological models, which typically focus on a “single pollutant–single target–single effect” paradigm, and instead enables a multidimensional and comprehensive assessment of toxic effects. The theoretical foundation of network toxicology is based on the “toxic network theory,” which proposes that environmental pollutants exert their effects not through isolated targets but by perturbing interconnected molecular networks – such as signaling pathways, metabolic circuits, and protein–protein interaction networks – ultimately leading to cellular dysfunction and disease phenotypes.^[13]^ This characteristic makes network toxicology particularly well suited for investigating the relationship between complex environmental exposures and multifactorial diseases.

Given the limited understanding of the direct molecular mechanisms, core targets, and regulatory pathways through which BPA influences cervical cancer, this study integrates network toxicology analysis with molecular docking validation. By combining systematic network-level analyses with precise molecular interaction modeling, we aim to provide mechanistic evidence elucidating how environmental exposure to BPA contributes to cervical cancer pathogenesis.

## 2. Methods

### 2.1. Network analysis of BPA toxicity

To obtain toxicity information on bisphenol A, we retrieved the SMILES structural formula (CC(C)(C1 = CC = C(C = C1)O)C2 = CC = C(C = C2)O) and molecular structure of the compound from the PubChem database (https://pubchem.ncbi.nlm.nih.gov/), and exported 2D and 3D images of bisphenol A from this database. Subsequently, the SMILES structure was imported into the ProTox-3.0 platform (https://tox.charite.de/protox3/), and the prediction results were integrated to evaluate the toxicity profile of BPA.

### 2.2. Targeted collection of bisphenol A

Using Chemical Biology Laboratory Database (ChEMBL, https://www.ebi.ac.uk/chembl/), the similarity ensemble method (SEA, https://sea.bkslab.org/), Target Network Prediction Database (TargetNet, http://targetnet.scbdd.com/), and Swiss target prediction (STP, http://www.swisstargetprediction.ch/), we predicted BPA targets. The selection criteria were as follows: targets annotated by Homo sapiens; STP probability score ≥0.5 (high confidence); ChEMBL activity value (IC50/Ki) ≤10 μM. Predictions with low confidence (probability <0.5) and nonhuman targets were excluded. Gene names were normalized using UniProt, and duplicates were removed to construct a library of BPA-associated targets.

### 2.3. Screening of cervical cancer-related targets

First, gene expression datasets related to cervical cancer were obtained from the Gene Expression Omnibus (GEO) database (https://www.ncbi.nlm.nih.gov/geo/). The datasets were normalized and analyzed for differential expression using the R programming language. Differentially expressed genes (DEGs) between cervical cancer tissues and normal control samples were identified, thereby expanding the pool of cervical cancer-related targets.

Subsequently, WGCNA was performed to explore gene expression patterns within the GEO dataset and to identify co-expression modules closely associated with the pathological characteristics of cervical cancer. Key module genes derived from the WGCNA analysis were integrated with the DEGs identified from GEO. Intersection analysis of gene sets from different sources, including GEO-derived DEGs and WGCNA key module genes, was conducted using Venn diagram analysis to obtain candidate cervical cancer-related targets.

### 2.4. Protein–protein interaction network analysis and core target screening

The overlapping target genes shared by BPA and cervical cancer were imported into the STRING database (version 12.0; https://string-db.org/), with the species restricted to Homo sapiens, to construct a PPI network. The resulting interaction data were then imported into Cytoscape software (version 3.10.3) for visualization and further network analysis.

Topological properties of network nodes and edges – including degree, weighted degree, closeness centrality, and betweenness centrality – were calculated to evaluate the relative importance of each protein within the network. Core targets were subsequently screened based on the following criteria: closeness centrality greater than the median value; radiality exceeding the median value; and degree and weighted degree both exceeding the median. The top-ranked core targets identified using these criteria were selected for subsequent molecular docking analysis.

### 2.5. Functional and pathway enrichment analysis of potential targets

Functional and pathway enrichment analyses were performed for the screened target genes associated with cervical cancer and BPA using gene ontology (GO), https://www.geneontology.org/) and the Kyoto encyclopedia of genes and genomes (KEGG; https://www.genome.jp/kegg/). GO enrichment analysis was initially conducted to characterize the biological functions of these target genes across 3 categories: biological processes (BP), cellular components (CC), and molecular functions (MF). Subsequently, KEGG pathway enrichment analysis was performed to identify signaling pathways potentially involved in BPA-induced cervical carcinogenesis.

The top 10 and top 5 enriched GO terms, as well as the top 10 and top 5 KEGG pathways, were ranked in descending order according to–log10 (*P*-value). Corresponding FDR values were annotated, and the results were visualized using bar plots and bubble charts. The “pheatmap” and “ggplot2” packages in R were employed to generate graphical representations of the GO and KEGG enrichment results, with both adjusted p-values and FDR values clearly displayed for each enriched term or pathway. Enrichment significance was assessed using a hypergeometric test, and *P*-values were corrected for multiple comparisons using the Benjamini–Hochberg method, with an FDR threshold set at < .05.

### 2.6. Correlation analysis between molecular docking and core targets in cervical cancer

Molecular docking analysis was conducted using CB-Dock2 to systematically investigate the binding interactions between BPA and the predicted core target proteins. The 2-dimensional chemical structure of BPA was retrieved from the PubChem database and saved in SDF(Structure Data File) format, which was subsequently converted to PDB(Protein Data Bank) format using Open Babel software (version 2.4.1). CB-Dock2, a blind docking platform, was applied to automatically identify potential binding cavities through curvature-based cavity detection, thereby determining the optimal binding site location and size.

CB-Dock2 is integrated with AutoDock Vina (version 1.2.3), which enhances the accuracy of binding site recognition and binding conformation prediction. For each ligand–protein complex, 3 independent docking runs were performed. Docking poses with a RMSD of <2.0 Å relative to the lowest-energy conformation were retained for further analysis.

**Figure 1.**
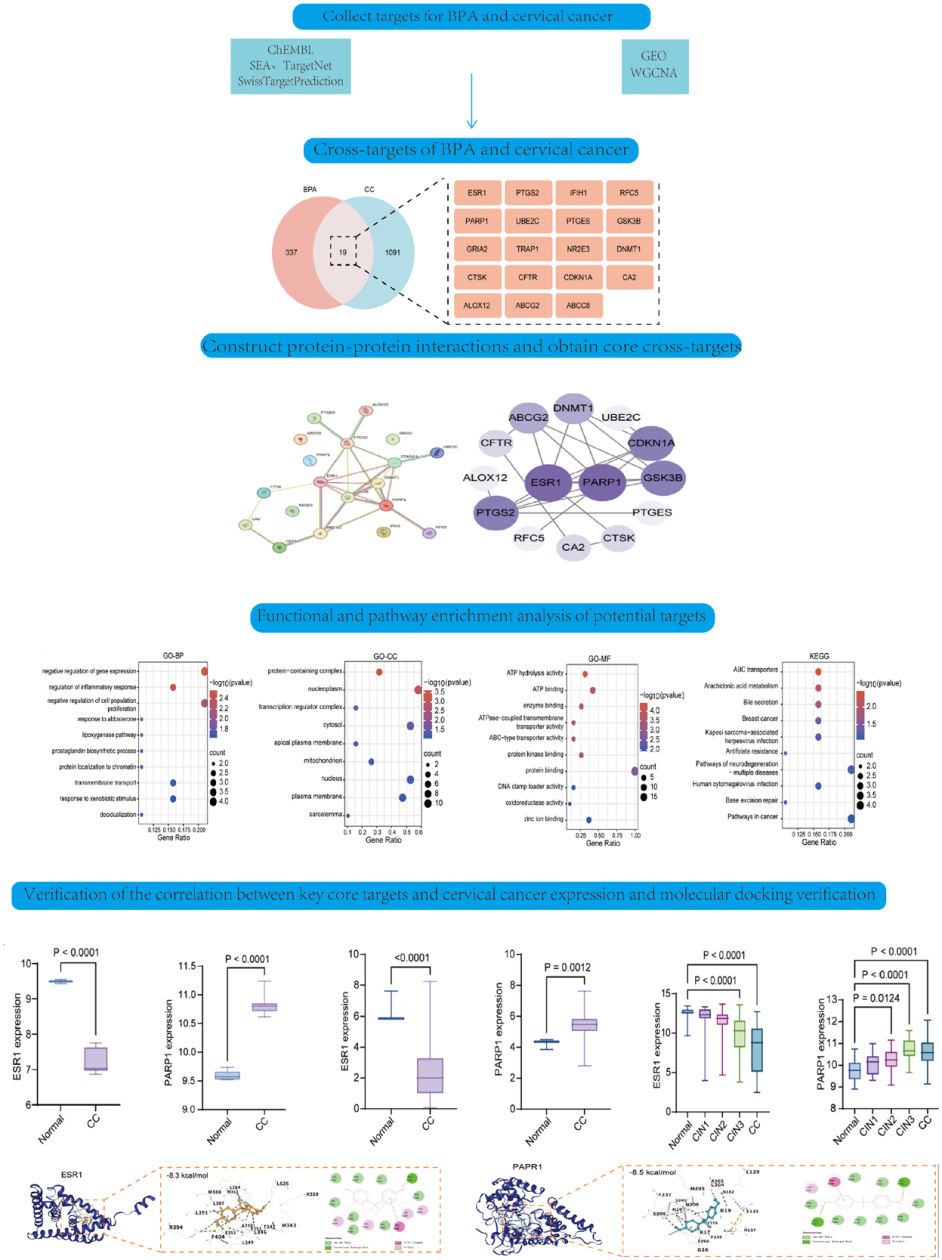
The complete experimental process is shown.

To validate the docking results, the corresponding protein crystal structures were retrieved from the Protein Data Bank (PDB, https://www.rcsb.org/) and the Computational Biology Laboratory (CLab) database (https://cbdm.uni-mainz.de/) for comparative analysis. All docking experiments were repeated 3 times under identical conditions to ensure reproducibility. In addition, the association between core targets and cervical cancer was verified using gene expression data from the GSE122697, TCGA, and GSE63514 datasets. The overall experimental workflow is illustrated in Figure 1.

## 3. Results

### 3.1. Toxicity evaluation and target acquisition of BPA

**Figure 2.**
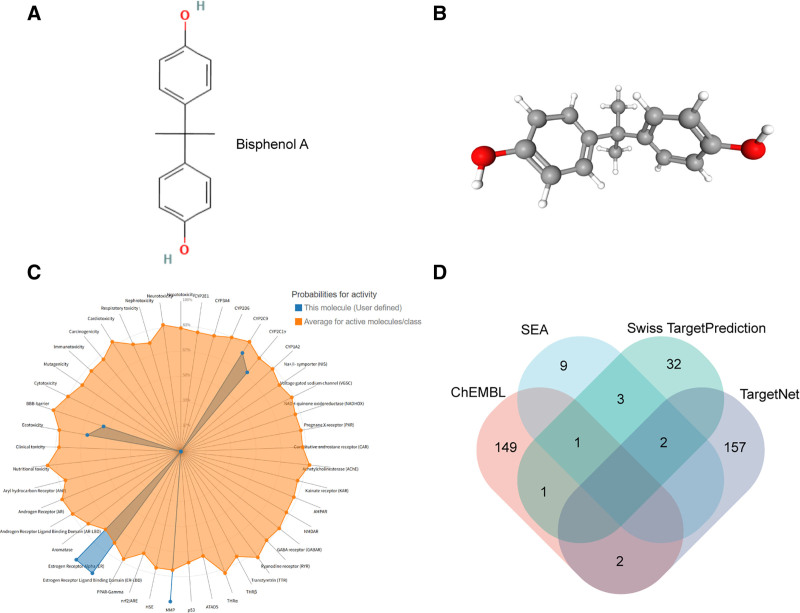
(A and B) BPA SMILES structure (CC(C)(C1 = CC = C(C = C1)O)C2 = CC = C(C = C2)O) retrieved from PubChem, with 2D and 3D structures generated. (C) BPA toxicity profile evaluated by ProTox-3.0 database, mainly showing endocrine and MMP toxicity. (D) Venn diagram of BPA targets from ChEMBL, TargetNet, SEA and SwissTargetPrediction; unique and overlapping targets are indicated. BPA = bisphenol A, CC = cellular components, ChEMBL = ChEMBL = chemical biology laboratory database, MMP = matrix metalloproteinase, SEA = similarity ensemble approach, SwissTargetPrediction = Swiss target prediction database, TargetNet = target network prediction database.

In this study, we obtained the SMILES structure of BPA from the PubChem database (CC(C)(C1 = CC = C(C = C1)O)C2 = CC = C(C = C2)O), and generated 2D and 3D images (Fig. 2A and B). Toxicity assessment was performed using the ProTox-3.0 database, which provides a comprehensive evaluation of the toxicological profile of BPA (Fig. 2C). The analysis indicated that BPA primarily exhibits endocrine-related toxicity and MMP (matrix metalloproteinase)–mediated tissue-damaging toxicity.

Target identification was subsequently conducted using multiple prediction databases. A total of 149 targets were uniquely identified in ChEMBL, 157 in TargetNet, 9 in SEA, and 32 in SwissTargetPrediction. In addition, 3 targets were shared across all 4 databases, 1 target overlapped between ChEMBL and SEA, and 2 targets overlapped between SwissTargetPrediction and TargetNet (Fig. 2D).

### 3.2. Acquisition of cervical cancer targets

**Figure 3.**
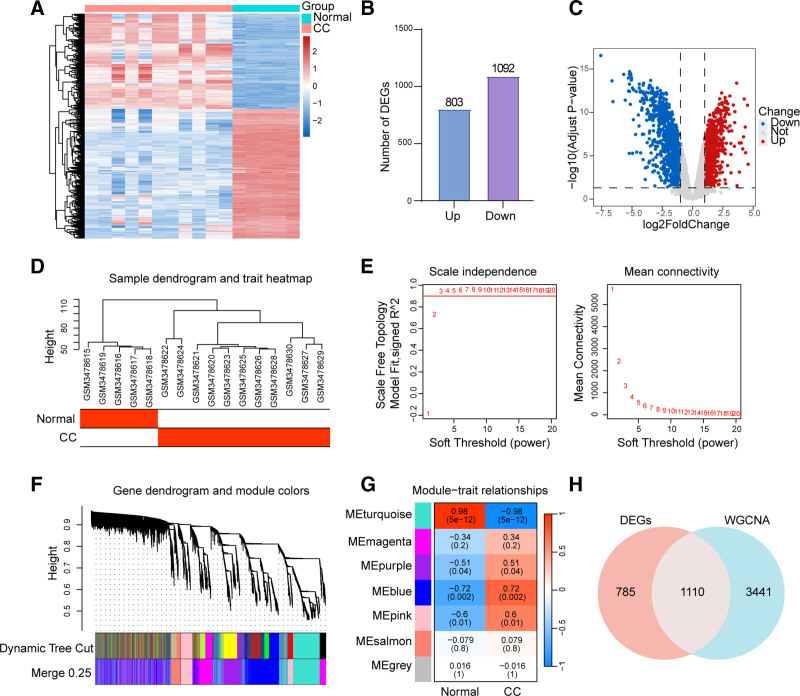
(A) Gene expression profiles of normal and cervical cancer groups with clear cluster separation, indicating altered gene expression during cervical cancer development. (B) 803 significantly up-regulated and 1092 down-regulated genes identified. (C) Volcano plot of differential genes (log_2_FoldChange ≥1, Adjusted *P* <.05); red: up-regulated, blue: down-regulated. (D) Sample clustering tree and trait heatmap validating grouping rationality, supporting subsequent analysis. (E) WGCNA soft-threshold selection; scale-free network (signed *R*^2^ = 0.9) ensuring analysis reliability. (F) Gene clustering tree and module division, facilitating module-phenotype association analysis. (G) Module-trait association heatmap; MEturquoise module strongly correlated with normal (*R* = 0.98, *P* = 5 × 10^−12^) and cervical cancer groups (*r* = −0.98, *P* = 5 × 10^−12^, key for cervical cancer. (H) Venn diagram showing 1110 overlapping genes between differential genes and WGCNA modules (core candidate targets for cervical cancer mechanisms). ME = module Eigengene, WGCNA = weighted correlation network analysis.

Analysis of gene expression profiles from normal control and cervical cancer samples revealed clear clustering separation between the 2 groups, indicating substantial transcriptional alterations during cervical cancer development (Fig. 3A). A total of 803 significantly up-regulated genes and 1092 significantly down-regulated genes were identified (Fig. 3B). Volcano plot analysis further facilitated the visualization and identification of DEGs. Genes were screened using thresholds of |log_2_ fold change| ≥1 and adjusted *P* <.05, yielding a robust set of statistically significant DEGs. In the volcano plot, red dots represent significantly up-regulated genes, whereas blue dots indicate significantly down-regulated genes (Fig. 3C).

The sample clustering dendrogram and trait heatmap demonstrated the validity of the sample grouping, with samples from the same group clustering together, reflecting high intragroup consistency in gene expression patterns and providing a reliable foundation for downstream analyses (Fig. 3D). In the WGCNA analysis, soft-threshold selection indicated that when the power parameter was chosen such that the signed scale-free topology fit index (R^2^) approached 0.9 and average connectivity stabilized, the constructed co-expression network conformed to scale-free network properties, ensuring the robustness of the analysis (Fig. 3E). Gene clustering dendrograms and module color assignments grouped genes with similar expression patterns into distinct modules, forming the basis for subsequent module-trait relationship analysis (Fig. 3F).

The module-trait relationship heatmap revealed several modules significantly associated with cervical cancer phenotypes. Notably, the MEturquoise (ME: Module Eigengene) module showed a strong positive correlation with the normal group (*R* = 0.98, *P* = 5 × 10^−12^) and a strong negative correlation with the cervical cancer group (*r* = −0.98, *P* = 5 × 10^−12^), suggesting that genes within this module may play a critical role in cervical carcinogenesis (Fig. 3G). Integration of differential expression analysis with WGCNA using Venn diagram analysis identified 1110 overlapping genes between the DEGs and the MEturquoise module (Fig. 3H). These genes were not only significantly differentially expressed but also belonged to the core co-expression module, representing key candidate targets for elucidating the molecular mechanisms underlying cervical cancer.

### 3.3. Functional and pathway enrichment analysis of potential targets

**Figure 4.**
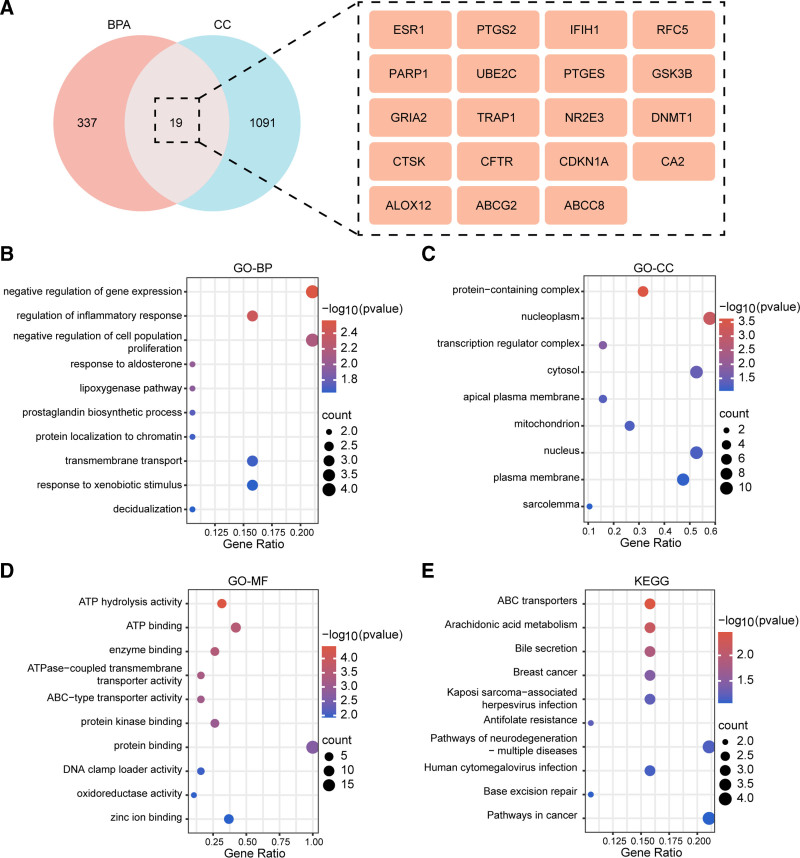
(A) Venn diagram of BPA- and cervical cancer-related targets; 337 BPA-specific, 1091 cervical cancer-specific, and 19 common targets. (B) GO-BP enrichment of common targets (abscissa: gene ratio; ordinate: BP terms; dot color: -log10(P-value); dot size: gene count). (C) GO-CC enrichment of common targets, mainly enriched in proteinaceous extracellular matrix, nucleoplasm, etc. (D) GO-MF enrichment of common targets (abscissa: gene ratio; ordinate: MF terms), significantly enriched in ATPase activity, ATP binding, etc. (E) KEGG pathway enrichment of common targets (abscissa: gene ratio; ordinate: pathways), notably enriched in ABC transporters, pathways in cancer, etc. ABC = ATP-binding cassette, BPA = bisphenol A, GO-BP = gene ontology–biological process, GO-CC = gene ontology–cellular component, GO-MF = gene ontology–molecular function, KEGG = Kyoto encyclopedia of genes and genomes.

Venn diagram analysis revealed the overlap between BPA-related targets and cervical cancer-related targets, identifying 337 BPA-specific targets, 1091 cervical cancer-specific targets, and 19 common targets shared by both (Fig. 4A). GO biological process (GO-BP) enrichment analysis of the common targets was visualized using bubble plots, in which the abscissa represents the gene ratio, the ordinate denotes enriched biological process terms, the color gradient corresponds to the negative logarithm of the adjusted *P*-value (–log_10_ *P*), and the bubble size reflects the number of enriched genes.

GO cellular component (GO-CC) enrichment analysis indicated that the common targets were primarily enriched in cellular structures such as the proteinaceous extracellular matrix, nucleoplasm, transcription factor complex, and cytoplasm, suggesting close functional associations with these cellular compartments (Fig. 4B and C). GO molecular function (GO-MF) enrichment analysis demonstrated significant enrichment in functions including ATPase activity (adenosine triphosphatase activity), ATP binding, enzyme binding, and ABC (ATP-binding cassette) transporter activity (Fig. 4D).

KEGG pathway enrichment analysis further revealed that the common targets were significantly enriched in pathways such as ABC transporters, arachidonic acid metabolism, bile secretion, and pathways in cancer, highlighting their potential involvement in BPA-related cervical cancer pathogenesis (Fig. 4E).

### 3.4. Identification of key core targets, validation of their association with cervical cancer, and molecular docking analysis

**Figure 5.**
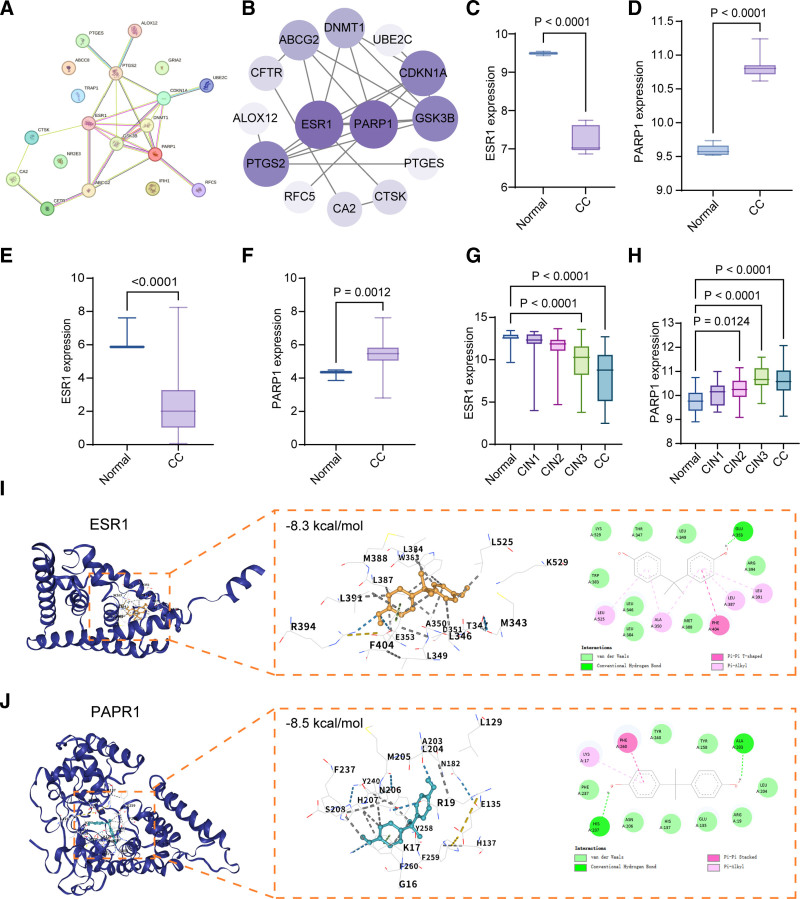
(A) PPI network of core targets (nodes: targets; connections: interaction relationships), providing topological basis for synergistic regulatory mechanism. (B) Core subnetwork of key targets; ESR1 and PARP1 are prominent. (C and D) GSE122697 dataset verification; ESR1 down-regulated (log_2_FC = −2.8, *P* <.0001), PARP1 up-regulated (log_2_FC = 3.1, *P* <.0001) in cervical cancer versus normal. (E and F) TCGA database verification; ESR1 (log_2_FC = -2.6, *P* < .0001) and PARP1 (log_2_FC = 2.9, *P* = .0012) expression differences consistent with previous results. (G and H) GSE63514 dataset analysis; ESR1 decreased, PARP1 increased with cervical lesion progression (cervical cancer vs CIN1: log_2_FC = −4.2/−4.5, *P* <.0001). (I) BPA-ESR1 molecular docking; stable binding with key amino acids (L384, etc.), hydrogen bond 2.5 to 3.5 Å, binding free energy −8.3 kcal/mol (strong affinity). (J) BPA-PARP1 molecular docking; stable binding with key amino acids (Y240, etc.), hydrogen bond 2.7 to 3.2 Å, binding free energy −8.5 kcal/mol (strong affinity). BPA = bisphenol A, CIN = cervical intraepithelial neoplasia, ESR1 = estrogen receptor 1, PARP1 = poly (ADP-ribose) polymerase 1, PPI = protein–protein interaction, TCGA = the cancer genome atlas.

The PPI network of the core targets is illustrated in Figure 5A. In this network, nodes of different colors represent distinct target proteins, while edges indicate their interaction relationships. This visualization highlights the interaction patterns among the co-targets shared by BPA and cervical cancer, providing a topological framework for understanding their potential synergistic regulatory mechanisms. A core subnetwork constructed from key targets further emphasizes the central roles of ESR1 and poly(ADP-ribose) polymerase 1 (PARP1), which emerged as the most prominent nodes within the network (Fig. 5B).

Expression analysis revealed significant differences in ESR1 and PARP1 expression between normal cervical tissues and cervical cancer samples. In the GSE122697 dataset, ESR1 expression was markedly down-regulated in cervical cancer compared with normal tissues (log_2_FC = −2.8, 95% CI: −3.2 to − 2.4, *P* < .0001), whereas PARP1 expression was significantly up-regulated (log_2_FC = 3.1, 95% CI: 2.7 to 3.5, *P* < .0001), preliminarily confirming aberrant expression patterns of these core targets in cervical cancer (Fig. 5C and D). These findings were further validated using TCGA database, which demonstrated consistent expression trends (ESR1: log_2_FC = −2.6, 95% CI: −3.0 to − 2.2, *P* <.0001; PARP1: log_2_FC = 2.9, 95% CI: 2.5 to 3.3, *P* = .0012), supporting the robustness and reliability of the observed expression differences (Fig. 5E and F).

To further explore the association between these targets and disease progression, expression changes in ESR1 and PARP1 across different stages of cervical lesions – CIN1, CIN2, CIN3 (cervical intraepithelial neoplasia grades 1–3) and cervical cancer – were analyzed using the GSE63514 dataset. ESR1 expression progressively decreased with lesion advancement (log_2_FC for cervical cancer vs CIN1 = −4.2, 95% CI: −4.7 to − 3.7, *P* <.0001), whereas PARP1 expression showed a gradual increase during disease progression (log_2_FC for cervical cancer vs CIN1 = 4.5, 95% CI: 4.0–5.0, *P* <.0001). Significant expression differences were already evident at the CIN stages, suggesting potential roles for these genes in early cervical carcinogenesis (Fig. 5G and H).

Molecular docking analysis demonstrated that BPA forms a stable complex with ESR1 by interacting with key amino acid residues at the active site of the 3-dimensional protein structure, including L384, W383, and E353. Hydrogen bond distances ranged from 2.5 to 3.5 Å, with a binding free energy of − 8.3 kcal/mol and a ligand efficiency of − 0.49 kcal/mol/atom, indicating a strong binding affinity. The interaction map further illustrated that BPA binds to ESR1 through a combination of hydrophobic interactions and hydrogen bonding (Fig. 5I).

Similarly, docking analysis between BPA and PARP1 revealed stable interactions with critical active-site residues, including Y240, H207, and R19. Hydrogen bond distances ranged from 2.7 to 3.2 Å, and the calculated binding free energy was − 8.5 kcal/mol, with a ligand efficiency of − 0.50 kcal/mol/atom, reflecting a strong affinity between BPA and PARP1. The interaction diagram confirmed that BPA binds to PARP1 through both hydrophobic interactions and hydrogen bonds (Fig. 5J).

## 4. Discussion

In this study, authoritative databases were first used to identify and analyze BPA-related and cervical cancer-related targets. Through integrated network toxicology analysis, 2 core targets – PARP1 and ESR1 – were identified. Gene Ontology (GO) and KEGG enrichment analyses were subsequently performed to explore the biological functions and signaling pathways associated with the shared targets. Furthermore, gene expression differences of these core targets were validated using the GSE63514 dataset and TCGA database across normal cervical tissues, CIN1, CIN2, CIN3, and cervical cancer tissues. Finally, molecular docking analyses were conducted using structural data from the PubChem database, OpenBabel software (version 2.4.1), the CB-Dock2 blind docking platform, and AutoDock Vina (version 1.2.3) to investigate the intermolecular interactions between BPA and the identified core target proteins. Collectively, these analyses provide insight into the potential molecular mechanisms through which BPA may contribute to cervical cancer development.

BPA is a widely used industrial chemical employed in the manufacture of plastics and epoxy resins.^[14]^ In recent years, increasing evidence has linked BPA exposure to cervical carcinogenesis.^[15]^ Previous studies have shown that low concentrations of BPA, acting as an environmental estrogen, can accelerate cervical epithelial cell proliferation and inhibit apoptosis.^[16]^ Other investigations have reported that although BPA does not directly induce cytotoxicity in cervical epithelial cells, high-dose exposure significantly increases the secretion of pro-inflammatory cytokines such as IL-6 (interleukin-6), which may lead to cervical tissue damage and adversely affect pregnancy outcomes.^[9]^ Together, these findings suggest a potential association between BPA exposure and the initiation and progression of cervical cancer, although the underlying molecular mechanisms remain incompletely understood.

PARP1 functions as a critical “molecular sensor” in the DNA damage response, detecting DNA strand breaks and facilitating DNA repair through the recruitment of repair proteins and the catalysis of ADP-ribosylation, thereby maintaining genomic stability.^[17–19]^ Aberrant activation of PARP1 has been closely associated with cervical carcinogenesis, and the Val762Ala polymorphism of the PARP1 gene has been shown to significantly increase cervical cancer risk, particularly among HPV(human papillomavirus)-positive individuals.^[20,21]^ Although direct evidence linking BPA exposure to PARP1 activation is currently limited, our network toxicology and molecular docking analyses revealed significant differential expression of PARP1 between normal cervical tissues and cervical cancer tissues, suggesting a potential functional association. These findings require further experimental validation in future studies.

Previous research has demonstrated that BPA can induce genomic instability through microRNA dysregulation.^[22]^ In this context, overactivation of PARP1 may represent a compensatory cellular response to DNA damage; however, persistent activation of this pathway may ultimately promote mutation accumulation and malignant transformation. In addition, BPA has been reported to activate cancer stem cell–like properties,^[23]^ which may intersect with PARP1-regulated pathways involved in DNA repair, cell survival, and therapy resistance.^[24]^ Based on the potential interaction between BPA exposure and PARP1 signaling, future studies should focus on 2 key aspects. First, it is essential to determine whether BPA exposure reduces the sensitivity of cervical cancer to conventional therapies by activating PARP1-mediated DNA repair pathways. Second, the therapeutic potential of PARP inhibitors in BPA-related cervical cancer warrants investigation, particularly in patients with documented environmental exposure. Targeting PARP1 may help reverse BPA-induced malignant phenotypes by blocking aberrant DNA repair processes, thereby offering new avenues for personalized treatment strategies.

ESR1, a key mediator of estrogen signaling, plays an important role in the pathological progression of cervical cancer.^[25]^ Numerous studies have demonstrated that ESR1 expression levels in cervical cancer tissues are closely associated with tumor stage and degree of differentiation.^[26]^ In cervical cancer cells, ESR1 can promote cellular proliferation and inhibit apoptosis by binding to estrogen-responsive elements within the promoter regions of target genes, thereby regulating the expression of cell cycle–related genes (such as Cyclin D1) and apoptosis-related genes (such as Bcl-2:B-cell lymphoma 2).^[27,28]^

In addition, aberrant ESR1 expression may influence the invasive and metastatic potential of cervical cancer. This effect is thought to be mediated, at least in part, through the regulation of EMT (epithelial–mesenchymal transition),^[29]^ a process closely associated with MMP activity.^[30]^ These findings are consistent with the results of our enrichment analyses and provide a molecular basis for understanding the mechanisms underlying cervical cancer development.

Although direct evidence linking BPA exposure to ESR1-mediated signaling in cervical cancer is currently limited, our network toxicology and molecular docking analyses suggest that BPA may interact with ESR1, thereby potentially disrupting normal estrogen signaling pathways. These findings warrant further experimental validation in future studies. Notably, BPA is known to exert biological effects through both canonical and noncanonical estrogen signaling pathways and may modulate ESR1 expression or activity in certain cervical cancer contexts.^[31,32]^

Future research should focus on 2 key directions. First, it is necessary to determine whether BPA exposure alters the sensitivity of cervical cancer to ESR1-targeted therapies by modulating ESR1 expression or function. Second, combined intervention strategies – such as simultaneously reducing BPA exposure and inhibiting ESR1 signaling – should be explored to develop more precise and personalized treatment approaches for cervical cancer patients with a history of environmental pollutant exposure.

## 5. Research limitations and prospects

Although gene expression data in this study were standardized, differences in sample sources and detection platforms across datasets may have introduced batch effects, potentially affecting the consistency and robustness of the results. In addition, network toxicology analysis relies on target predictions derived from existing databases, which may exclude low-confidence targets and fail to capture currently unknown targets. These limitations could restrict the scope of the inferred associations between BPA and cervical cancer.

Moreover, the principal conclusions of this study are based primarily on bioinformatics analyses and molecular docking simulations. The absence of experimental validation – including in vitro assays (such as verification of ESR1 and PARP1 expression following BPA exposure in cervical cancer cell lines) and in vivo animal model studies – precludes definitive confirmation of a causal relationship between BPA exposure and cervical carcinogenesis mediated through ESR1, PARP1, and related signaling pathways.

Future studies should therefore incorporate cervical cancer cell models and BPA-exposed animal models to validate these findings. The expression changes of ESR1 and PARP1 could be confirmed using Western blotting and RT–PCR(reverse transcription–polymerase chain reaction), while the effects of BPA on cervical cancer cell proliferation, apoptosis, and invasion could be systematically evaluated using flow cytometry and Transwell assays. Such experiments would help clarify the causal mechanisms underlying BPA-induced cervical carcinogenesis.

In addition, analyses of clinical samples are warranted to investigate the relationship between BPA exposure levels, ESR1 and PARP1 expression, and clinicopathological characteristics of cervical cancer. Future research should also consider the combined effects of multiple environmental endocrine disruptors to assess potential synergistic regulatory influences on cervical cancer-related targets and pathways. Integration of single-cell sequencing technologies may further elucidate cell type–specific responses to BPA exposure and reveal more precise molecular mechanisms.

Finally, given the central roles of ESR1 and PARP1 identified in this study, future work should explore the therapeutic potential of PARP inhibitors and ESR1 modulators in BPA-associated cervical cancer, with the goal of developing personalized treatment strategies for patients with documented environmental exposure. Concurrently, the development of early detection biomarkers for BPA exposure may facilitate early screening and timely diagnosis of cervical cancer.

## 6. Conclusion

In this study, the molecular mechanisms underlying BPA–mediated cervical cancer were systematically investigated using an integrated approach combining network toxicology and molecular docking analysis. The results indicated that BPA primarily exhibits endocrine-related toxicity and MMP–mediated tissue-damaging toxicity. BPA-associated targets were identified through multi-database prediction and subsequent standardization. Differential expression analysis of GEO datasets, together with WGCNA, enabled the identification of core cervical cancer-related genes, yielding 19 common targets shared between BPA and cervical cancer.

Functional enrichment analyses revealed that these common targets were significantly enriched in BP and signaling pathways, including the negative regulation of gene expression and cancer-related pathways, suggesting that BPA may contribute to cervical carcinogenesis through modulation of these mechanisms. Protein–protein interaction network analysis further identified ESR1 and PARP1 as central core targets. Validation using multiple independent datasets (GSE122697, TCGA, and GSE63514) demonstrated that ESR1 was significantly down-regulated, whereas PARP1 was significantly up-regulated in cervical cancer tissues, with expression changes correlating with the progression of cervical lesions.

Molecular docking analysis showed that BPA exhibited stable binding to ESR1 and PARP1, with binding free energies of − 8.3 kcal/mol and − 8.5 kcal/mol, respectively. Collectively, these findings suggest that BPA may promote cervical cancer development by interacting with ESR1 and PARP1 to regulate key oncogenic pathways. These targets may serve as potential diagnostic biomarkers and therapeutic intervention points in cervical cancer. However, as the present conclusions are based solely on bioinformatics analyses and molecular docking simulations, further experimental validation is required to substantiate these findings.

## Author contributions

**Conceptualization:** Long Yang.

**Formal analysis:** Yutai Zhao.

**Validation:** Wanjun Zhang, Yuqian Zhu, Hongli Dong, Long Yang.

**Visualization:** Long Yang.

**Writing – original draft:** Yi Li, Wanjun Zhang, Lin Tang, Yuqian Zhu, Hongli Dong, Yutai Zhao.

**Writing – review & editing:** Yutai Zhao.
